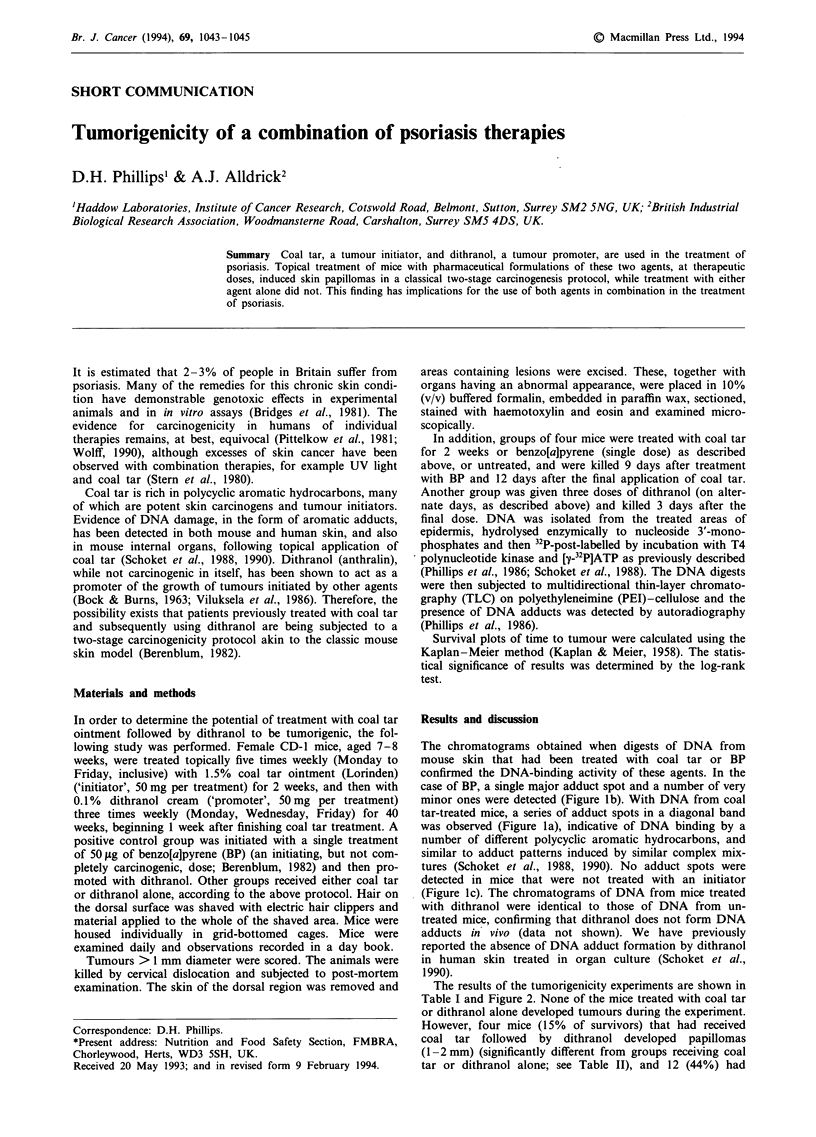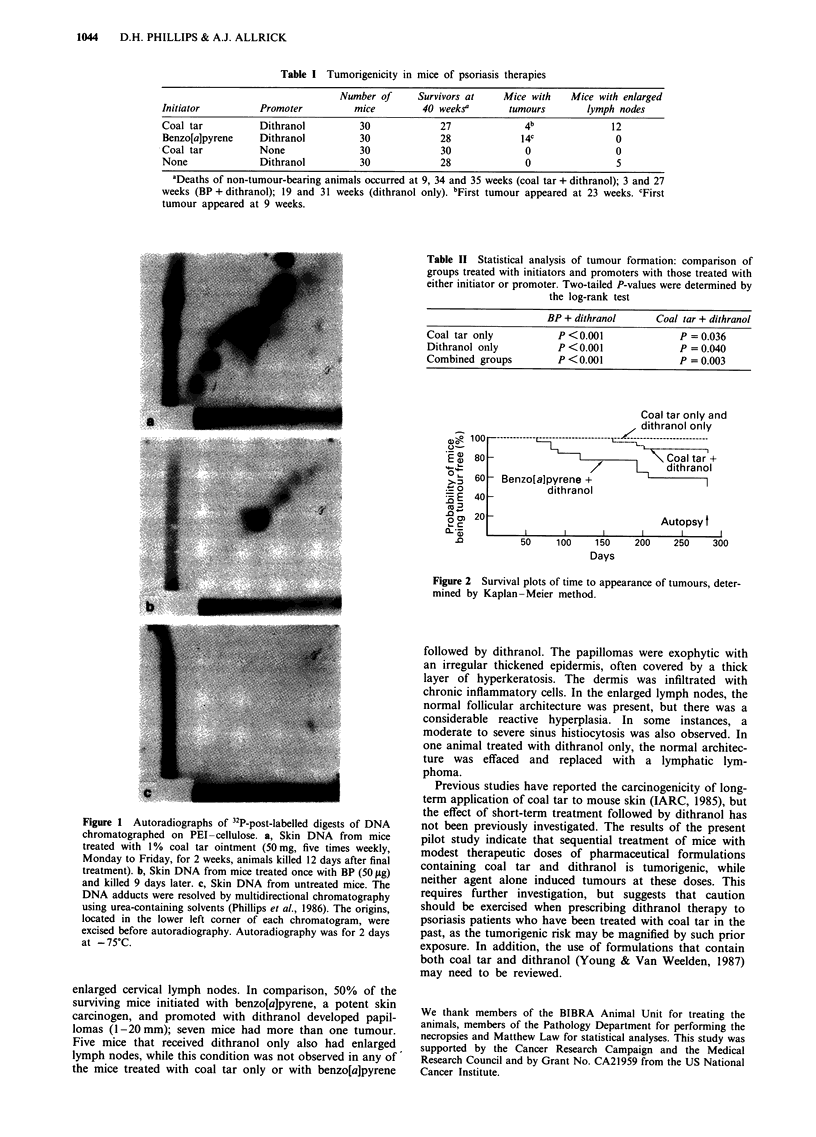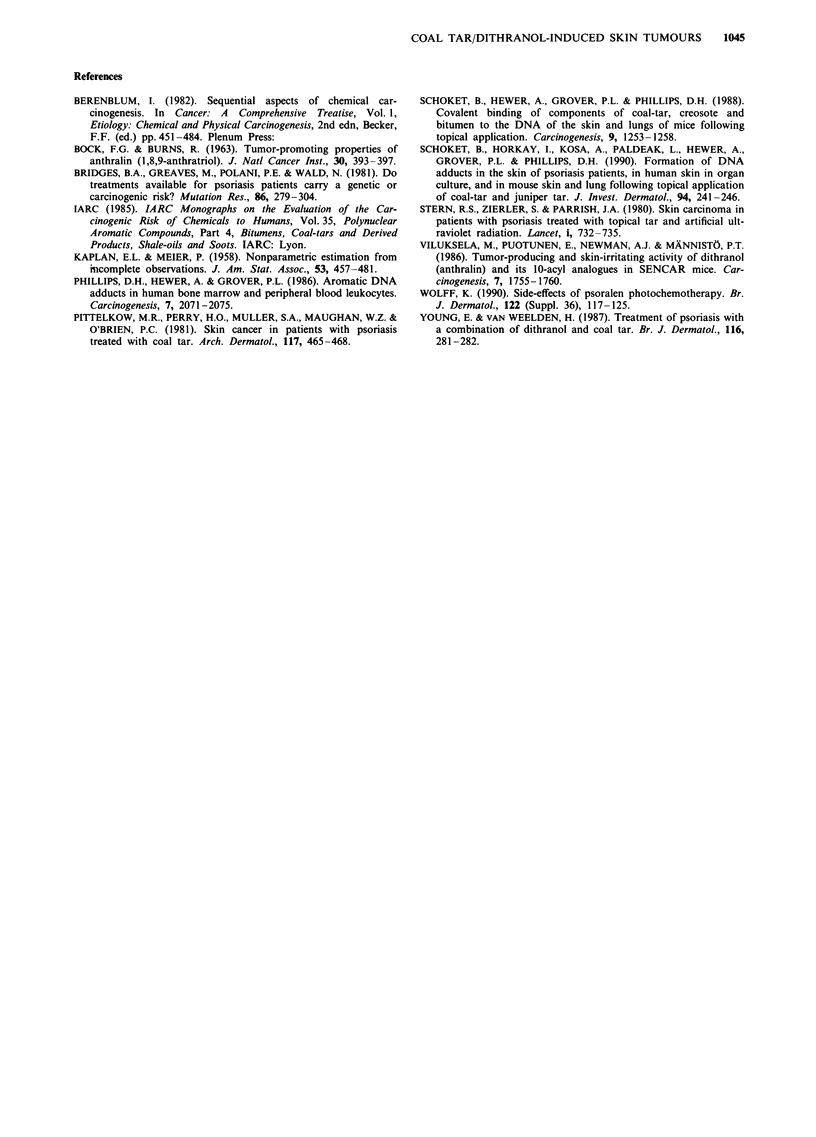# Tumorigenicity of a combination of psoriasis therapies.

**DOI:** 10.1038/bjc.1994.205

**Published:** 1994-06

**Authors:** D. H. Phillips, A. J. Alldrick

**Affiliations:** Haddow Laboratories, Institute of Cancer Research, Belmont, Sutton, Surrey, UK.

## Abstract

**Images:**


Br. J. Cancer (1994), 69, 1043–1045
© Macmillan Press Ltd., 1994

SHORT COMMUNICATION

### Tumorigenicity of a combination of psoriasis therapies

D.H. Phillips[1] & A.J. Alldrick[2]

[1]Haddow Laboratories, Institute of Cancer Research, Cotswold Road, Belmont, Sutton, Surrey SM2 5NG, UK; [2]British Industrial Biological Research Association, Woodmansterne Road, Carshalton, Surrey SM5 4DS, UK.

**Summary** Coal tar, a tumour initiator, and dithranol, a tumour promoter, are used in the treatment of psoriasis. Topical treatment of mice with pharmaceutical formulations of these two agents, at therapeutic doses, induced skin papillomas in a classical two-stage carcinogenesis protocol, while treatment with either agent alone did not. This finding has implications for the use of both agents in combination in the treatment of psoriasis.

It is estimated that 2–3% of people in Britain suffer from psoriasis. Many of the remedies for this chronic skin condition have demonstrable genotoxic effects in experimental animals and in *in vitro* assays (Bridges *et al.*, 1981). The evidence for carcinogenicity in humans of individual therapies remains, at best, equivocal (Pittelkow *et al.*, 1981; Wolff, 1990), although excesses of skin cancer have been observed with combination therapies, for example UV light and coal tar (Stern *et al.*, 1980).

Coal tar is rich in polycyclic aromatic hydrocarbons, many of which are potent skin carcinogens and tumour initiators. Evidence of DNA damage, in the form of aromatic adducts, has been detected in both mouse and human skin, and also in mouse internal organs, following topical application of coal tar (Schoket *et al.*, 1988, 1990). Dithranol (anthralin), while not carcinogenic in itself, has been shown to act as a promoter of the growth of tumours initiated by other agents (Bock & Burns, 1963; Viluksela *et al.*, 1986). Therefore, the possibility exists that patients previously treated with coal tar and subsequently using dithranol are being subjected to a two-stage carcinogenicity protocol akin to the classic mouse skin model (Berenblum, 1982).

### Materials and methods

In order to determine the potential of treatment with coal tar ointment followed by dithranol to be tumorigenic, the following study was performed. Female CD-1 mice, aged 7–8 weeks, were treated topically five times weekly (Monday to Friday, inclusive) with 1.5% coal tar ointment (Lorinden) ('initiator', 50 mg per treatment) for 2 weeks, and then with 0.1% dithranol cream ('promoter', 50 mg per treatment) three times weekly (Monday, Wednesday, Friday) for 40 weeks, beginning 1 week after finishing coal tar treatment. A positive control group was initiated with a single treatment of 50 μg of benzo[*a*]pyrene (BP) (an initiating, but not completely carcinogenic, dose; Berenblum, 1982) and then promoted with dithranol. Other groups received either coal tar or dithranol alone, according to the above protocol. Hair on the dorsal surface was shaved with electric hair clippers and material applied to the whole of the shaved area. Mice were housed individually in grid-bottomed cages. Mice were examined daily and observations recorded in a day book.

Tumours > 1 mm diameter were scored. The animals were killed by cervical dislocation and subjected to post-mortem examination. The skin of the dorsal region was removed and areas containing lesions were excised. These, together with organs having an abnormal appearance, were placed in 10% (v/v) buffered formalin, embedded in paraffin wax, sectioned, stained with haemotoxylin and eosin and examined microscopically.

In addition, groups of four mice were treated with coal tar for 2 weeks or benzo[*a*]pyrene (single dose) as described above, or untreated, and were killed 9 days after treatment with BP and 12 days after the final application of coal tar. Another group was given three doses of dithranol (on alternate days, as described above) and killed 3 days after the final dose. DNA was isolated from the treated areas of epidermis, hydrolysed enzymically to nucleoside 3'-monophosphates and then $^{32}P$-post-labelled by incubation with T4 polynucleotide kinase and [$\gamma$-$^{32}P$]ATP as previously described (Phillips *et al.*, 1986; Schoket *et al.*, 1988). The DNA digests were then subjected to multidirectional thin-layer chromatography (TLC) on polyethyleneimine (PEI)–cellulose and the presence of DNA adducts was detected by autoradiography (Phillips *et al.*, 1986).

Survival plots of time to tumour were calculated using the Kaplan–Meier method (Kaplan & Meier, 1958). The statistical significance of results was determined by the log-rank test.

### Results and discussion

The chromatograms obtained when digests of DNA from mouse skin that had been treated with coal tar or BP confirmed the DNA-binding activity of these agents. In the case of BP, a single major adduct spot and a number of very minor ones were detected (Figure 1b). With DNA from coal tar-treated mice, a series of adduct spots in a diagonal band was observed (Figure 1a), indicative of DNA binding by a number of different polycyclic aromatic hydrocarbons, and similar to adduct patterns induced by similar complex mixtures (Schoket *et al.*, 1988, 1990). No adduct spots were detected in mice that were not treated with an initiator (Figure 1c). The chromatograms of DNA from mice treated with dithranol were identical to those of DNA from untreated mice, confirming that dithranol does not form DNA adducts *in vivo* (data not shown). We have previously reported the absence of DNA adduct formation by dithranol in human skin treated in organ culture (Schoket *et al.*, 1990).

The results of the tumorigenicity experiments are shown in Table I and Figure 2. None of the mice treated with coal tar or dithranol alone developed tumours during the experiment. However, four mice (15% of survivors) that had received coal tar followed by dithranol developed papillomas (1–2 mm) (significantly different from groups receiving coal tar or dithranol alone; see Table II), and 12 (44%) had

Correspondence: D.H. Phillips.
*Present address: Nutrition and Food Safety Section, FMBRA, Chorleywood, Herts, WD3 5SH, UK.
Received 20 May 1993; and in revised form 9 February 1994.

**Table I** Tumorigenicity in mice of psoriasis therapies

| *Initiator* | *Promoter* | *Number of mice* | *Survivors at 40 weeks*[a] | *Mice with tumours* | *Mice with enlarged lymph nodes* |
|---|---|---|---|---|---|
| Coal tar | Dithranol | 30 | 27 | 4[b] | 12 |
| Benzo[*a*]pyrene | Dithranol | 30 | 28 | 14[c] | 0 |
| Coal tar | None | 30 | 30 | 0 | 0 |
| None | Dithranol | 30 | 28 | 0 | 5 |

[a]Deaths of non-tumour-bearing animals occurred at 9, 34 and 35 weeks (coal tar + dithranol); 3 and 27 weeks (BP + dithranol); 19 and 31 weeks (dithranol only). [b]First tumour appeared at 23 weeks. [c]First tumour appeared at 9 weeks.

**Figure 1** Autoradiographs of $^{32}P$-post-labelled digests of DNA chromatographed on PEI-cellulose. **a**, Skin DNA from mice treated with 1% coal tar ointment (50 mg, five times weekly, Monday to Friday, for 2 weeks, animals killed 12 days after final treatment). **b**, Skin DNA from mice treated once with BP (50 μg) and killed 9 days later. **c**, Skin DNA from untreated mice. The DNA adducts were resolved by multidirectional chromatography using urea-containing solvents (Phillips *et al.*, 1986). The origins, located in the lower left corner of each chromatogram, were excised before autoradiography. Autoradiography was for 2 days at −75°C.

**Table II** Statistical analysis of tumour formation: comparison of groups treated with initiators and promoters with those treated with either initiator or promoter. Two-tailed *P*-values were determined by the log-rank test

| | *BP + dithranol* | *Coal tar + dithranol* |
|---|---|---|
| Coal tar only | $P < 0.001$ | $P = 0.036$ |
| Dithranol only | $P < 0.001$ | $P = 0.040$ |
| Combined groups | $P < 0.001$ | $P = 0.003$ |

**Figure 2** Survival plots of time to appearance of tumours, determined by Kaplan-Meier method.

enlarged cervical lymph nodes. In comparison, 50% of the surviving mice initiated with benzo[*a*]pyrene, a potent skin carcinogen, and promoted with dithranol developed papillomas (1–20 mm); seven mice had more than one tumour. Five mice that received dithranol only also had enlarged lymph nodes, while this condition was not observed in any of the mice treated with coal tar only or with benzo[*a*]pyrene followed by dithranol. The papillomas were exophytic with an irregular thickened epidermis, often covered by a thick layer of hyperkeratosis. The dermis was infiltrated with chronic inflammatory cells. In the enlarged lymph nodes, the normal follicular architecture was present, but there was a considerable reactive hyperplasia. In some instances, a moderate to severe sinus histiocytosis was also observed. In one animal treated with dithranol only, the normal architecture was effaced and replaced with a lymphatic lymphoma.

Previous studies have reported the carcinogenicity of long-term application of coal tar to mouse skin (IARC, 1985), but the effect of short-term treatment followed by dithranol has not been previously investigated. The results of the present pilot study indicate that sequential treatment of mice with modest therapeutic doses of pharmaceutical formulations containing coal tar and dithranol is tumorigenic, while neither agent alone induced tumours at these doses. This requires further investigation, but suggests that caution should be exercised when prescribing dithranol therapy to psoriasis patients who have been treated with coal tar in the past, as the tumorigenic risk may be magnified by such prior exposure. In addition, the use of formulations that contain both coal tar and dithranol (Young & Van Weelden, 1987) may need to be reviewed.

We thank members of the BIBRA Animal Unit for treating the animals, members of the Pathology Department for performing the necropsies and Matthew Law for statistical analyses. This study was supported by the Cancer Research Campaign and the Medical Research Council and by Grant No. CA21959 from the US National Cancer Institute.

### References

BERENBLUM, I. (1982). Sequential aspects of chemical carcinogenesis. In *Cancer: A Comprehensive Treatise*, Vol. 1, *Etiology: Chemical and Physical Carcinogenesis*, 2nd edn, Becker, F.F. (ed.) pp. 451-484. Plenum Press:

BOCK, F.G. & BURNS, R. (1963). Tumor-promoting properties of anthralin (1,8,9-anthratriol). *J. Natl Cancer Inst.*, **30**, 393-397.

BRIDGES, B.A., GREAVES, M., POLANI, P.E. & WALD, N. (1981). Do treatments available for psoriasis patients carry a genetic or carcinogenic risk? *Mutation Res.*, **86**, 279-304.

IARC (1985). *IARC Monographs on the Evaluation of the Carcinogenic Risk of Chemicals to Humans*, Vol. 35, *Polynuclear Aromatic Compounds*, Part 4, *Bitumens, Coal-tars and Derived Products, Shale-oils and Soots*. IARC: Lyon.

KAPLAN, E.L. & MEIER, P. (1958). Nonparametric estimation from incomplete observations. *J. Am. Stat. Assoc.*, **53**, 457-481.

PHILLIPS, D.H., HEWER, A. & GROVER, P.L. (1986). Aromatic DNA adducts in human bone marrow and peripheral blood leukocytes. *Carcinogenesis*, **7**, 2071-2075.

PITTELKOW, M.R., PERRY, H.O., MULLER, S.A., MAUGHAN, W.Z. & O'BRIEN, P.C. (1981). Skin cancer in patients with psoriasis treated with coal tar. *Arch. Dermatol.*, **117**, 465-468.

SCHOKET, B., HEWER, A., GROVER, P.L. & PHILLIPS, D.H. (1988). Covalent binding of components of coal-tar, creosote and bitumen to the DNA of the skin and lungs of mice following topical application. *Carcinogenesis*, **9**, 1253-1258.

SCHOKET, B., HORKAY, I., KOSA, A., PALDEAK, L., HEWER, A., GROVER, P.L. & PHILLIPS, D.H. (1990). Formation of DNA adducts in the skin of psoriasis patients, in human skin in organ culture, and in mouse skin and lung following topical application of coal-tar and juniper tar. *J. Invest. Dermatol.*, **94**, 241-246.

STERN, R.S., ZIERLER, S. & PARRISH, J.A. (1980). Skin carcinoma in patients with psoriasis treated with topical tar and artificial ultraviolet radiation. *Lancet*, **i**, 732-735.

VILUKSELA, M., PUOTUNEN, E., NEWMAN, A.J. & MÄNNISTÖ, P.T. (1986). Tumor-producing and skin-irritating activity of dithranol (anthralin) and its 10-acyl analogues in SENCAR mice. *Carcinogenesis*, **7**, 1755-1760.

WOLFF, K. (1990). Side-effects of psoralen photochemotherapy. *Br. J. Dermatol.*, **122** (Suppl. 36), 117-125.

YOUNG, E. & VAN WEELDEN, H. (1987). Treatment of psoriasis with a combination of dithranol and coal tar. *Br. J. Dermatol.*, **116**, 281-282.